# Supplementary material for: A Systematic Review and Meta-Analysis of Enzyme Replacement Therapy in Late-Onset Pompe Disease
Source: J Clin Med. 2021 Oct 21;10(21):4828. doi: 10.3390/jcm10214828 (PMC8584814; doi:10.3390/jcm10214828)
Supplement: Supplementary file 1 [file jcm-10-04828-s001.zip › jcm-1384124-supplementary.pdf]

BACK MATTER

Table S1: Evaluation of the effect of enzyme replacement therapy on forced vital capacity in late-onset Pompe disease

Study	Patients (n)	Pre-ERT FVC μ (sd)	Post-ERT FVC μ (sd)	Mean change (p-value)
Angelini et al. (2012)	68	65.2 (26.5)	66.5 (26.6)	N/A (p=0.22)
de Vries et al. (2012)	69	68.3 (median)	N/A	0.10 (95% CI -1 to 1.1; p=0.92)
Furusawa et al. (2012)	5	16.02 (17.71)	23.46 (24.5)	7.44 (95% CI -1.04 to 15.92)
Kuperus et al. (2017)	84	72.0	73.5	N/A
Montagnese et al. (2015)	13	71.69 (16.1)	68.0 (17.36)	-3.69 (95% CI -9.16 to 1.77)
Regnery et al. (2012)	28	80.27 (14.08)	77.19 (18.05)	-1.50 (95% CI -4.11 to 1.1)
Van Capelle et al. (2010)	5	68.49 (24.43)	71.83 (18.99)	3.34 (95% CI -2.48 to 9.16)
Van der Ploeg et al. (2012)	53	55.4 (14.4)	57.2 (16.2)	0.80 (95% CI -1.1 to 2.6)
Van der Ploeg et al. (2016)	15	76.4 (15.63)	77.6 (28.46)	1.80 (95% CI -7.3 to 10.9; p=0.67)
Vianello et al. (2013) A	8	37.0 (14.09)	37.86 (12.72)	0.86 (95% CI -1.26 to 2.97; p=0.48)

*FVC=Forced vital capacity. ERT=enzyme replacement therapy. sd=standard deviation. CI=confidence interval. N/A=not available.

Table S2: GRADEpro of forced vital capacity outcome

Certainty assessment						
**Participants (studies) Follow up**	**Risk of bias**	**Inconsistency**	**Indirectness**	**Imprecision**	**Publication bias**	**Overall certainty of evidence**
348 (10 observational studies) Mean 33.8 months	very serious [a]	not serious	serious [e]	not serious	publication bias strongly suspected [d]	⊕◯◯◯ VERY LOW

a. No randomization or control group; d. Studies sponsored by the pharmaceutical industry; e. Secondary outcome.

Table S3: Evaluation of the effect of enzyme replacement therapy on 6-minute walking test in late-onset Pompe disease

Study	Patients (n)	Distance walked in the 6MWT in meters Pre-ERT μ (sd)	Distance walked in the 6MWT in meters Post-ERT μ (sd)	Mean change (p-value)
Angelini et al. (2009)	5	305.82 (174.64)	341.0 (163.77)	N/A
Angelini et al. (2012)	58	320.0 (161.0)	383.0 (178.0)	N/A (p<0.0001)
Bembi et al. (2010) Adult	17	226.07 (192.77)	261.35 (190.52)	35.28 (95% CI -1.75 to 72.31; p=0.00)
Bembi et al. (2010) Juvenile	7	421.95 (283.3)	614.33 (233.1)	192.38 (95% CI 29.44 to 355.32)
Kuperus et al. (2017)	53			N/A (p=0.03)
Montagnese et al. (2015)	14	323.17 (144.46)	327.67 (144.77)	4.50 (95% CI -16.97 to 25.97)

Ravaglia et al. (2010)	11	245.8 (185.3)	294.8 (194.4)	N/A (p=0.00)
Ravaglia et al. (2012)	7	339.29 (156.67)	342.91 (157.89)	3.63 (95% CI -8.54 to 15.79)
Regnery et al. (2012)	21	312.0 (165.5)	325.6 (174.8)	-39.61 (95% CI -68.05 to -11.18; p=0.49)
Van Capelle et al. (2010)	5	454.32 (85.66)	582.48 (42.85)	128.16 (95% CI 83.89 to 172.43)
Van der Ploeg et al. (2012)	53	332.2 (126.7)	358.3 (150.1)	21.30 (95% CI -0.20 to 42.80)
Van der Ploeg et al. (2016)	15	449.9 (208.01)	471.2 (223.6)	37.30 (95% CI 7.70 to 67.00; p=0.02)
Witkowski et al. (2018)	5	507.0 (14.6)	606.0 (6.8)	19.50

*6MWT= 6-minute walking test. ERT=enzyme replacement therapy. sd=standard deviation. CI=confidence interval. N/A=not available.

Table S4: GRADEpro of 6-minute walking test outcome

**Certainty assessment**						
**Participants (studies) Follow up**	**Risk of bias**	**Inconsistency**	**Indirectness**	**Imprecision**	**Publication bias**	**Overall certainty of evidence**
271 (12 observational studies) Mean 36 months	very serious [a]	very serious [b]	serious [e]	not serious	publication bias strongly suspected strong association [d]	⊕◯◯◯ VERY LOW

a. No randomization or control group; b. Heterogeneity between studies, without controlling for confounding factors; d. Studies sponsored by the pharmaceutical industry; e. Secondary outcome.

Table S5: Evaluation of the effect of enzyme replacement therapy on Walton & Gardner-Medwin scale scores in late-onset Pompe disease

**Study**	**Patients (n)**	**Pre-ERT μ (sd)**	**Post-ERT μ (sd)**	**Association measures**
Angelini et al. (2012)	68	N/A	N/A	Improvement in 18/68 patients (26%); p=0.22
Montagnese et al. (2015)	14	5.5 (2.4)	5.5 (2.4)	N/A
Orlikowski et al. (2011)	5	N/A	N/A	No improvement (data not shown)
Regnery et al. (2012)	38	4.4 (2.1)	12 mo after= 4.1 (2.4)<br>24 mo after= 4.1 (2.3)<br>36 mo after= 4.4 (2.4)	p=n.s.
Strothotte et al. (2010)	44	N/A	N/A	Improvement in 4/44 (%) p=0.074
Vianello et al. (2013)	Group A: 8<br>Group B: 6	Group A= 5.1 (1.9)<br>Group B- 6.1 (1.9)	Group A= 5.7 (1.2)<br>Group B= 6.1 (1.9)	p=0.33

*ERT=Enzyme replacement therapy. sd=standard deviation. N/A=not available. n.s.=not significant.

Table S6: GRADEpro of Walton & Gardner-Medwin Scale outcome

Certainty assessment						
**Participants (studies) Follow up**	**Risk of bias**	**Inconsistency**	**Indirectness**	**Imprecision**	**Publication bias**	**Overall certainty of evidence**
183 (6 observational studies) Median 33.4 months	serious [a]	not serious [b]	serious	serious [c]	publication bias strongly suspected [d]	⊕◯◯◯ VERY LOW

* a. No randomization or control group; b. Heterogeneity between studies, without controlling for confounding factors; c. No effect measure evaluation; d. Studies sponsored by the pharmaceutical industry.

Table S7: Evaluation of the effect of enzyme replacement therapy on upper limb strength in late-onset Pompe disease

**Study**	**Patients (n)**	**Outcome**	**Pre-ERT μ (sd)**	**Post-ERT μ (sd) or pp/year**	**Association measures (95% CI)**
de Vries et al. (2012)	69	MRC *sum score*; QMFT HHD	N/A	MRC *sum score:* + 1.4 pp/year QMFT: +0.7 pp/year HHD: +4 pp/year	MRC *sum score:* 0.8 to 2.1; p <0.001 QMFT: -0.2 to 1.7; p = 0.14 HHD: 2.5 to 5.6; p <0.001
Kuperus et al. (2017)	88	MRC *sum score*; QMFT *sum scores*; dynamometry	N/A	MRC + 6.6 pp; QMFT *sum scores* +1.5 pp Dynamometry + 9.6 pp	MRC p<0.0001; QMFT p=0.47; Dynamometry p<0.001
Papadimas et al. (2011)	5	MRC *sum score*	28	29	N/A
Regnery et al. (2012)	38	MRC *sum score*	42.29 (8.49)	12 mo after= 41.92 (8.62); 24 mo after= 43.89 (5.16); 36 mo after= 41.19 (7.61)	p=n.s.
Strothotte et al. (2010)	44	MRC *sum score*	41.5 (5.7) (median 43.3)	42 (6.5) (median 44.8)	p = 0.317
van Capelle et al. (2010)	5	QMFT HHD	QMFT: 75.9 HHD: 649.5	QMFT: 94.3 HHD: 1096	QMFT: p = 0.04 HHD: p = 0.01
van der Ploeg et al. (2010)	90	QMT-*Arm score*	alglucosidase alfa: 55.9% placebo: 56.9%	alglucosidase alfa, week 78: 60.9% placebo, week 78: 58.3%; treatment effect estimate: 3.57%	p = 0.19
van der Ploeg et al. (2012)	60	QMT-*Arm score*	55.9 (20.4)	61.1 (21.4) (+4.3)	N/A
van der Ploeg et al. (2016)	16	QMFT upper and lower segment dynamometry	QMFT: 44.5 (11.8) Upper limb dynamometry: 2065.5 (859.8)	QMFT: 46.8 (11.3) (+ 5.3%) Upper limb dynamometry: 2108.7 (850.7) (+4.7)	QMFT: p = 0.045 Upper limb dynamometry: p = 0.39

*ERT=Enzyme replacement therapy. sd=standard deviation. pp=percentage points. CI=confidence interval. MRC=Medical Research Council. HHD=handheld dynamometry. QMFT=Quick Motor Function Test. N/A=not available. n.s.=not significant.

Table S8: GRADEpro of upper limb strength outcome (assessed with QMFT and HHD)

Certainty assessment						
**Participants (studies) Follow up**	**Risk of bias**	**Inconsistency**	**Indirectness**	**Imprecision**	**Publication bias**	**Overall certainty of evidence**
90 (3 observational studies); median 24,5 months	not serious [a]	serious [b]	serious [e]	not serious	publication bias strongly suspected [d]	⨁◯◯◯ VERY LOW

a. No randomization or control group; b. Heterogeneity between studies, without controlling for confounding factors; d. Studies sponsored by the pharmaceutical industry; e. Secondary outcome.

Table S9: Evaluation of the effect of enzyme replacement therapy on quality of life in late-onset Pompe disease

**Study**	**Patients (n)**	**Instrument**	**Value Pre-ERT μ (sd)**	**Value post-ERT μ (sd)**	**Association measures (95% CI)**
Gungor et al. (2015)	174	SF-36	Physical component: -0.73 (95% CI -1.07 to -0.39)<br>Mental component: 0.16 (95% CI -0.25 to 0.57)	Physical component: 0-2 years of ERT: 1.49; > 2 years: -0.15<br>Mental component: 0-2 years of ERT: 1.03; > 2 years: 0.02	Physical component: 0-2 years of ERT: p <0.01 (0.76 to 2.21);<br>> 2 years: p> 0.05 (-0.43 to 0.13)<br>Mental component: 0-2 years p> 0.05 (-0.07- 2.13);<br>> 2 years: p> 0.05 (-0.41 to 0.46)
Orlikowski et al. (2011)	5	SF-36	Physical component: 25.24 (6.69)<br>Mental component: 46.84 (18.43)	Physical component: 29.22 (3.50)<br>Mental component: 57.52 (17.33)	N/A
Regnery et al. (2012)	38	SF-36	50 (10)	No changes	p=n.s.
Strothotte et al. (2010)	44	SF-36	48.5	No changes	N/A
van der Ploeg et al. (2010)	90	SF-36	Alglucosidase alfa group: 34.3 (8.9)<br>Placebo group: 34.9 (7.3)	Alglucosidase alfa group: 35.1 (9.8), change 0.80 (95% CI −1.22 to - 2.82)<br>Placebo group: 36.5 (9.6), change 1.16 (95% CI −1.64 to - 3.97)	Difference between groups: 0.37 (−3.83 to - 3.09); p = 0.83
van der Ploeg et al. (2016)	15	Peds QL	58.5 (20.74) (12.5 to 87.5)	66 (15.73) (36.1 to 91.7)	Change from baseline 8.1 (17.01) (−1.4 to 17.5); p = 0.088

*ERT=Enzyme replacement therapy. sd=standard deviation. CI=confidence interval. N/A=not available. n.s.=not significant.

Table S10: GRADEpro of quality of life outcome (assessed with SF-36)

Certainty assessment						
**Participants (studies) Follow up**	**Risk of bias**	**Inconsistency**	**Indirectness**	**Imprecision**	**Publication bias**	**Overall certainty of evidence**
277 (4 observational studies) Median 24 months	serious [a]	serious [b]	serious [e]	not serious	publication bias strongly suspected [d]	⊕◯◯◯ VERY LOW

a. No randomization or control group; B. Heterogeneity between studies, without controlling for confounding factors; d. Studies sponsored by the pharmaceutical industry; e. Secondary outcome.

Table S11: Evaluation of the effect of enzyme replacement therapy on time on ventilation in late-onset Pompe disease

Study	Patients (n)	Pre-ERT (hours) μ (sd)	Post-ERT (hours) M (sd)	Association measures (95% CI)
Bembi et al. (2010)	24	14	12 mo after: 8	36 mo after: p <0.0001; 12 mo after: p = 0.0005
Angelini et al. (2012)*	21/74	15.6	12.1	p = 0.005
Regnery et al. (2012)	38	16.6 (7.1)	16.6 (7.1)	N/A
Vianello et al. (2013)	8	17.3 (3.1)	12.5 (7.6); Control: 19 (14.3)	p = 0.006; mean change: -4.8 (-8.2 to 1.5);<br>control: -0.16 (-4.5 to 3.7); p = 0.004

*Data presented for patients on ventilation since the beginning of the study (n=21). ERT=Enzyme replacement therapy. sd=standard deviation. CI=confidence interval. N/A=not available.

Table S12: GRADEpro of time on ventilation

Certainty assessment						
**Participants (studies) Follow up**	**Risk of bias**	**Inconsistency**	**Indirectness**	**Imprecision**	**Publication bias**	**Overall certainty of evidence**
117 (4 observational studies) Median 36 months	not serious [a]	serious [b]	serious [e]	not serious	publication bias strongly suspected [d] strong association	⊕⊕◯◯ LOW*

a. No randomization or control group; B. Heterogeneity between studies, without controlling for confounding factors; d. Studies sponsored by the pharmaceutical industry; e. Secondary outcome. *Due to large effect of intervention.

Table S13. Safety assessment of enzyme replacement therapy for patients with late-onset Pompe disease

Article	Outcome	N patients	N events
Angelini et al. (2012)	Death	74	1
	Any AE	74	4
	AB+	15	11
Bembi et al. (2010)	IAR	24	2
de Vries et al. (2012)	Any IAR	69	12
	Death	69	2
de Vries et al. (2017)	Ac +	73	46
	IAR	13	715
	Any IAR	73	13
Kuperus et al. (2017)	Any IAR	88	19
	Death	88	7
Orlikowski et al. (2011)	SAE	5	1
	AE	5	58
	AB+	5	5
Regnery et al. (2012)	Death	38	0
	AB+	38	38
Strothotte et al. (2010)	Death	44	0
van Capelle et al. (2010)	IAR	5	0
	AB+	5	5
van der Ploeg et al. (2010)	Any AE	60	13
	Any AE	30	6
	Death	60	1
	AB+	59	59
	IAR	60	17
	Any IAR	30	7
Van der Ploeg et al. (2012)	Any AE	60	60
	Any IAR	60	21
	Any SAE	60	15
	Death	60	0
	AB+	59	59
van der Ploeg et al. (2016)	SAE	16	1
	IAR	6	24
	Any IAR	16	6
	Death	16	0
Vianello et al. (2013)	SAE	8	0
	IAR	8	0
	Death	8	0
	Death	6	2

*AE=adverse event. SAE=serious adverse event. IAR= infusion-associated reaction. AB+=presence of anti-alglucosidase alfa antibodies.

Table S14: GRADEpro of safety outcomes

Certainty assessment

Participants (studies) Follow up	Risk of bias	Inconsistency	Indirectness	Imprecision	Publication bias	Overall certainty of evidence
540 (12 observational studies) Median 35 months	serious [a]	serious [b,e]	serious [e]	not serious	publication bias strongly suspected [d]	⊕◯◯◯ VERY LOW

a. No randomization or control group; B. Heterogeneity between studies, without controlling for confounding factors; d. Studies sponsored by the pharmaceutical industry; e. Secondary outcome.

Table S15: GRADEpro of mortality

Certainty assessment							Summary of findings				
Participants (studies) Follow up	Risk of bias	Inconsistency	Indirectness	Imprecision	Publication bias	Overall certainty of evidence	Study event rates (%)		Relative effect (95 % CI)	Anticipated absolute effects	
							With placebo	With alglucosidase alfa		Risk with placebo	Risk difference with alglucosidase alfa
										Low	
493 (9 observational studies)	serious [a]	not serious	serious [e]	not serious [c]	publication bias strongly suspected [d] strong association	⊕⊕◯◯ LOW *	2/36 (5.6 %)	11/457 (2.4%)	**RR 0.21** (0.11 to 0.41)	0 per 1.000	**0 fewer per 1.000** (from 0 fewer to 0 fewer)

CI=Confidence interval; RR=risk ratio. a. No randomization or control group; c. No effect measure evaluation. d. Studies sponsored by the pharmaceutical industry; e. Secondary outcome. *Due to large effect of intervention.
